# Iterative hypothesis testing in HIV vaccine research: moving towards success

**DOI:** 10.1002/jia2.26102

**Published:** 2023-05-17

**Authors:** Pervin Anklesaria, Nina D. Russell

**Affiliations:** ^1^ Bill & Melinda Gates Foundation Seattle Washington USA

1

The development of an HIV vaccine remains a critical goal for global health. New and emerging prevention methods, such as long‐acting pre‐exposure prophylaxis, may help reduce the risk of HIV acquisition, but it is widely accepted that a highly efficacious, durable and cost‐effective vaccine could accelerate the decline of HIV incidence in high‐burden countries. Twenty years ago, leaders in the field introduced the concept of a global HIV vaccine enterprise and called for a new collaborative approach to HIV vaccine research [1]. The resulting significant increase in funding, and structured collaborative networks, led to important advancements in the understanding of HIV envelope (Env) structural biology, novel approaches to immunogen design, the nature of protective immune responses and innovative delivery methods. Thus, the current imperative for the field is to synthesize the cumulative knowledge gained and use it to define specific critical hypotheses that can be methodically and rigorously tested through small, early‐phase, carefully designed experimental studies that can inform the selection of product candidates for efficacy evaluation.

The scientific challenges of developing a prophylactic vaccine against HIV are well known. HIV is a highly diverse virus that mutates rapidly and establishes latency early in the course of infection. The virus evades immune‐mediated defences against natural infection, and cases of stringent and durable immune control of virus replication are rare. Low HIV envelope spike density, obscured by a dense glycan shield, renders the HIV envelope protein poorly immunogenic compared to other virus envelope proteins [2]. To prevent established infection, an effective HIV vaccine will need to either block HIV acquisition entirely, and/or rapidly contain initial virus replication and abort primary HIV infection.

Apart from the modestly efficacious RV144 phase 3 trial [3], a study conducted in Thailand evaluating an ALVAC‐gag vector prime in combination with gp120 protein boost, each of the key large‐scale HIV vaccine studies conducted in recent years was stopped for lack of efficacy in preventing HIV acquisition [4]. The Uhambo (HVTN 702) phase 2b‐3 trial evaluated a recombinant canarypox vector (ALVAC‐HIV) containing subtype C envelope (vCP2438) with an MF59‐adjuvanted subtype C bivalent glycoprotein 120 (gp120) boost, and the phase 2b Imbokodo (HVTN 705) and phase 3 Mosaico (HVTN 706) studies evaluated “mosaic” immunogens—based on multiple HIV subtypes—administered as a serotype 26 adenovirus vector (Ad26.Mos4.HIV) with an aluminium phosphate‐adjuvanted gp140 boost. Each of these approaches was intended to trigger broader and more robust binding and functional antibody responses, as well as T‐cell responses, than earlier vaccine concepts. While the trials were successfully conducted and the vaccine regimens were safe and immunogenic, the resultant anti‐ENV binding (non‐neutralizing) antibodies and only moderate levels of CD4^+^ and class 1 restricted antigen‐specific CD8^+^ T cells, were insufficient to prevent HIV infection or rapidly kill HIV‐infected cells. There has been speculation that several key factors, such as insufficient binding antibody responses to the Env V1‐V2 region (identified as a correlate of risk in the RV144 trial), a higher force of infection and/or greater genetic diversity in the trial populations and, most importantly, a lack of bnAb responses, contributed to the lack of efficacy of these approaches. In parallel with the aforementioned vaccine trials, data from the “AMP” (antibody‐mediated prevention) efficacy studies [5], evaluating the administration of a bnAb, VRC01, showed that a single bnAb is able to prevent infection from only very sensitive circulating viral strains, providing a surrogate measure of Ab titres required for the prevention of HIV acquisition via vaccination. Similarly, non‐human primate studies have also shown that bnAbs can prevent simian‐human immunodeficiency virus (SHIV) infection in monkeys. Thus, while the diversity of HIV remains daunting, many groups are now focused on vaccine concepts designed to elicit potent and bnAb against conserved viral epitopes.

Recent articles have outlined novel strategies for inducing bnAbs with vaccines [6, 7]. Most employ sequential administration of different structure‐guided HIV‐Env‐based immunogens engineered to elicit cross‐reactive neutralizing Ab titres against bnAb epitopes through vaccination [8]. The identification of rationally designed immunogens for sequential administration, including priming immunogens that target bNab epitope B‐cell precursors, is expected to require an iterative process of evaluation in both pre‐clinical systems and small clinical studies. Recent advances in physiologically relevant mouse models will be critical to enabling faster iterative cycles for the selection of candidates [9, 10]. For bnAb‐inducing regimens, a systematic, precise, in‐depth evaluation of the responses stimulated by each immunogen in a sequential regimen will be required to assess if the needed rare bnAb B‐cell precursors are engaged, and antibodies undergo the somatic hypermutation and maturation required to generate epitope‐specific nAbs [11]. Thus, innovation and speed will also be needed in the analysis of vaccine‐elicited immune responses. And, it is clear from the AMP study, and modelling, that an effective vaccine will likely require high concentrations of potent nAbs against at least two or three bnAb epitopes. A deeper understanding of the molecular basis of somatic hypermutation and selection of high‐affinity antibody‐producing cells in the germinal centre (GC), as well as the factors that influence inter‐clonal competition of GC B cells, and re‐entry of memory B cells into GCs is crucial [12]. Finally, it will be vital to know how to generate long‐lived plasma cells that can secrete cross‐reactive nAbs for at least 5–10 years.

Studies with a rhesus cytomegalovirus (CMV)‐based vaccine have shown unprecedented levels of virus control following simian immunodeficiency virus (SIV) challenge in rhesus macaques through a different mechanism of protection, namely major histocompatability complex E (MHC‐E) restricted antigen‐specific CD8^+^ T cells (non‐classical) [13]. Further, Arunachalam et al. have demonstrated that a Class 1 restricted antigen‐specific CD8^+^ T‐cell (classical) vaccine response can lower the titres of neutralizing antibodies required to prevent a mucosal SHIV infection [14]. Thus, it is likely that a cross‐neutralizing Ab‐inducing vaccine regimen will also need to integrate components that elicit classical [15] or non‐classical CD8^+^ T cells [13] to prevent or clear HIV infection. A key remaining hypothesis to be tested in human efficacy studies is whether high‐frequency cytotoxic effector memory CD8^+^ T cells available at the site of virus entry, in combination with cross‐reactive neutralizing Ab titres against multiple bnAb epitopes, could block or suppress nascent HIV replication resulting in the prevention of persistent HIV infection.

Iterative, small, experimental clinical studies using novel delivery systems, such as mRNA, protein nanoparticles plus adjuvants, and/or viral vectors, will be essential for validating data from pre‐clinical studies. They provide a mechanism for generating incremental human data to advance vaccine immunogen discovery and demonstrate the feasibility of eliciting the aforementioned immune responses. Once a regimen is identified from these early‐phase studies, vaccine candidates can be advanced to more traditional product development trials with a higher probability of success. Given the likely requirement for multi‐component vaccine regimens, efforts are underway to develop pulsatile or controlled‐release technologies that can sequentially deploy multiple immunogens after a single immunization [16, 17]. Such innovative delivery solutions will be essential to ensuring practical and cost‐effective distribution of complex vaccine regimens in high‐burden countries.

The HIV vaccine field is currently emerging from years of effort focused on largely empiric, hypothesis‐generating phase 2b trials in the absence of known correlates of immune protection. Now is a critical time to leverage recent learnings and technological advancements to collectively articulate clear hypotheses for rationale immunogen design that can be systematically and rapidly addressed in a coordinated manner with early engagement with relevant communities and regulatory agencies.

## AUTHORS’ CONTRIBUTIONS

PA and NDR drafted, reviewed and revised the manuscript. Both authors approved the final version of the manuscript.

## COMPETING INTERESTS

The authors declare that they have no competing interests.

## DISCLAIMER

The views expressed are solely those of the authors and do not necessarily reflect the views of the Bill & Melinda Gates Foundation.
